# Hepatitis E Virus in 3 Types of Laboratory Animals, China, 2012–2015

**DOI:** 10.3201/eid2212.160131

**Published:** 2016-12

**Authors:** Lin Wang, Yulin Zhang, Wanyun Gong, William Tianshi Song, Ling Wang

**Affiliations:** Peking University Health Science Center, Beijing, China (Lin Wang, Y. Zhang, W. Gong, Ling Wang);; Beijing Huijia Private School, Beijing (W.T. Song)

**Keywords:** hepatitis E virus, HEV, viruses, laboratory animals, rabbits, monkeys, pigs, virus latency, China

## Abstract

We found seroprevalences for hepatitis E virus (HEV) of 7.5%, 18.5%, and 83.3% in specific pathogen-free (SPF) laboratory rabbits, monkeys, and pigs, respectively, in China. HEV RNA was detected in 4.8% of SPF rabbits, and 11 rabbits had latent infections. Screening for HEV in SPF animals before relevant experiments are conducted is recommended.

Hepatitis E virus (HEV) is a single-stranded, positive-sense RNA virus that belongs to the family *Hepeviridae* and is transmitted by the fecal–oral route (*1*). The lack of an efficient cell culture system for HEV hinders understanding of this pathogen. In most HEV studies, specific pathogen-free (SPF) animals are used (*2*). However, antibodies against HEV have been detected in 5 of 10 SPF rabbits in the United States (*3*). Antibodies against HEV or HEV RNA in laboratory animals will confound experimental results.

In addition, swine HEV is zoonotic to humans, and rabbit HEV-3 has been shown to be infectious to cynomolgus macaques (*4*). A strain of HEV closely related to rabbit HEV has been isolated from a human in France (*5*). These findings suggest that laboratory animals infected with HEV might put laboratory workers at risk for infection. In this study, we investigated the antibodies against HEV and HEV RNA in 3 types of SPF laboratory animals (monkeys, pigs and rabbits) that are commonly used in HEV studies in China.

## The Study

This study was approved by the Committee of Laboratory Animal Welfare and Ethics, Peking University Health Science Center. In 2012, we obtained 146 SPF rhesus monkeys (*Macaca mulatta*) and cynomolgus monkeys (*M. fascicularis*) from a commercial institute of biologic resources in Beijing, China. During 2012–2015, we obtained 332 SPF rabbits from 2 qualified vendors in China: supplier A (New Zealand white rabbits) and supplier B (Japanese white rabbits). We also obtained 6 SPF Bama miniature pigs from supplier B (Table 1). Microbes excluded in SPF animals are shown in Technical Appendix Table 1).

**Table 1:** Characteristics of laboratory rabbits, monkeys, and pigs tested for hepatitis E virus, China, 2012–2015*

Batch no.	Sampling year	Age	No. animals	Species	No. positive, PCR/antibodies against HEV			Genotype	Acceptability, %‡
					Total	First sampling	Subsequent sampling†		
1	2012	3 wk	10	NZW	0/0	0/0	0/0	NA	100
2	2012	6 wk	16	NZW	0/0	0/0	0/0	NA	100
3	2012	2.5 y	20	CM	0/18	0/18	0/0	NA	10
3	2012	2.5 y	126	RM	0/9	0/9	0/0	NA	92.9
4	2013	7 wk	21	NZW	0/3	0/3	0/0	NA	85.7
5	2013	7 wk	43	JW	0/0	0/0	0/0	NA	100
6	2013	28 wk	32	JW	0/4	0/4	0/0	NA	87.5
7	2014	12 wk	92	JW	2/10	2/10	0/0	3	87.0
8	2015	4 wk	6	BMP	0/5	0/5	0/0	NA	16.7
9	2015	12 wk	42	NZW	1/2	1/2	0/0	3	92.9
10	2015	12 wk	76	JW	6/13	4/4	2/9	3	75

*BMP, Bama miniature pig (*Sus scrofa domestica*); CM, cynomolgus monkey (*Macaca fascicularis*); HEV, hepatitis E virus; JW, Japanese white rabbit (*Oryctolagus cuniculus*); NA, not applicable; NZW, New Zealand white rabbit (*Oryctolagus cuniculus*); RM, rhesus monkey (*Macaca mulatta*). †Animals had negative results for PCR or antibodies against HEV at week 1, but results became positive for samples collected in subsequent weeks. ‡An animal was considered acceptable for the HEV study when all test results remained negative for 4 consecutive weeks.

All animals were bred in China and housed in polycarbonate individual ventilated cages or mini pig stainless steel cages (Suhang, Jiangsu, China). Paired serum and fecal samples were collected weekly from each animal for 4 consecutive weeks. We stopped sampling when we obtained positive results for antibodies against or HEV RNA. Specific procedures of for sample processing were described (*4*).

Serum samples from monkeys were tested by using human anti-HEV IgM and human anti-HEV IgG ELISA kits (Wantai, Beijing, China) (*6*). Serum samples from rabbits and pigs were tested by using an anti-HEV total antibodies ELISA kit (Wantai) and HEV E2 antigen (aa 394–606 of open reading frame 2) (*7*). Signal-to-cutoff values were calculated, and values >1 were considered positive.

Virus RNA was extracted from 100 μL of serum or 50% fecal suspensions by using TRIzol Reagent (Invitrogen, Burlington, Ontario, Canada). All samples were analyzed by using a nested reverse transcription PCR.

HEV-positive samples were sequenced and submitted to GenBank (accession nos. KU217460–KU217473 and KU218407–KU218408). A phylogenetic tree was constructed by using MEGA 6.0 software (*8*). A more detailed description of the complete protocol has been previously published (*4*).

We detected antibodies against HEV in 25 (7.5%) of 332 SPF rabbits and 5 (83.3%) of 6 SPF Bama miniature pigs. The HEV IgM–positive rate was 0% (0/146), and the HEV IgG–positive rate was 18.5% (27/146) for SPF monkeys (Table 2). Among all antibody-positive animals, HEV RNA was not detected in serum or stool samples. The HEV antibody–positive rate for SPF rabbits in China was lower than that for farmed and wild rabbits in other studies (Technical Appendix Table 2).

**Table 2:** Prevalence of antibodies against hepatitis E virus and virus RNA in laboratory animals, China, 2012–2015*

Animal, species	No. samples	No. (%) positive for antibodies against HEV				No. (%) positive for HEV RNA	
IgM	IgG	IgM + IgG	Serum	Stool
Rabbit							
NZW	89	NA	NA	5 (5.6)		1 (1.1)	1 (1.1)
JW	243	NA	NA	20 (8.2)		0 (0)	15 (6.2)
Total	332	NA	NA	25 (7.5)		1 (0.3)	16 (4.8)
Monkey							
RM	126	0 (0)	9 (7.1)	NA		0 (0)	0 (0)
CM	20	0 (0)	18 (90.0)	NA		0 (0)	0 (0)
Total	146	0 (0)	27 (18.5)	NA		0 (0)	0 (0)
Pig							
BMP	6	NA	NA	5 (83.3)		0 (0)	0 (0)

*BMP, Bama miniature pig (*Sus scrofa domestica*); CM, cynomolgus monkey (*Macaca fascicularis*); HEV, hepatitis E virus; JW, Japanese white rabbit (*Oryctolagus cuniculus*); NA, not applicable; NZW, New Zealand white rabbit (*Oryctolagus cuniculus*); RM, rhesus monkey (*Macaca mulatta*).

HEV RNA was detected in 16 (4.8%) of 332 SPF rabbits. One rabbit (supplier A, sequence no. 16) was viremic and shed virus in feces; the other 15 rabbits (supplier B, sequence nos. 1–15) only shed virus in feces (Table 2). Phylogenetic analysis confirmed that all strains isolated from the SPF rabbits belong to genotype 3 and are in 3 clusters for reported rabbit HEV strains (Figure).

**Figure:**
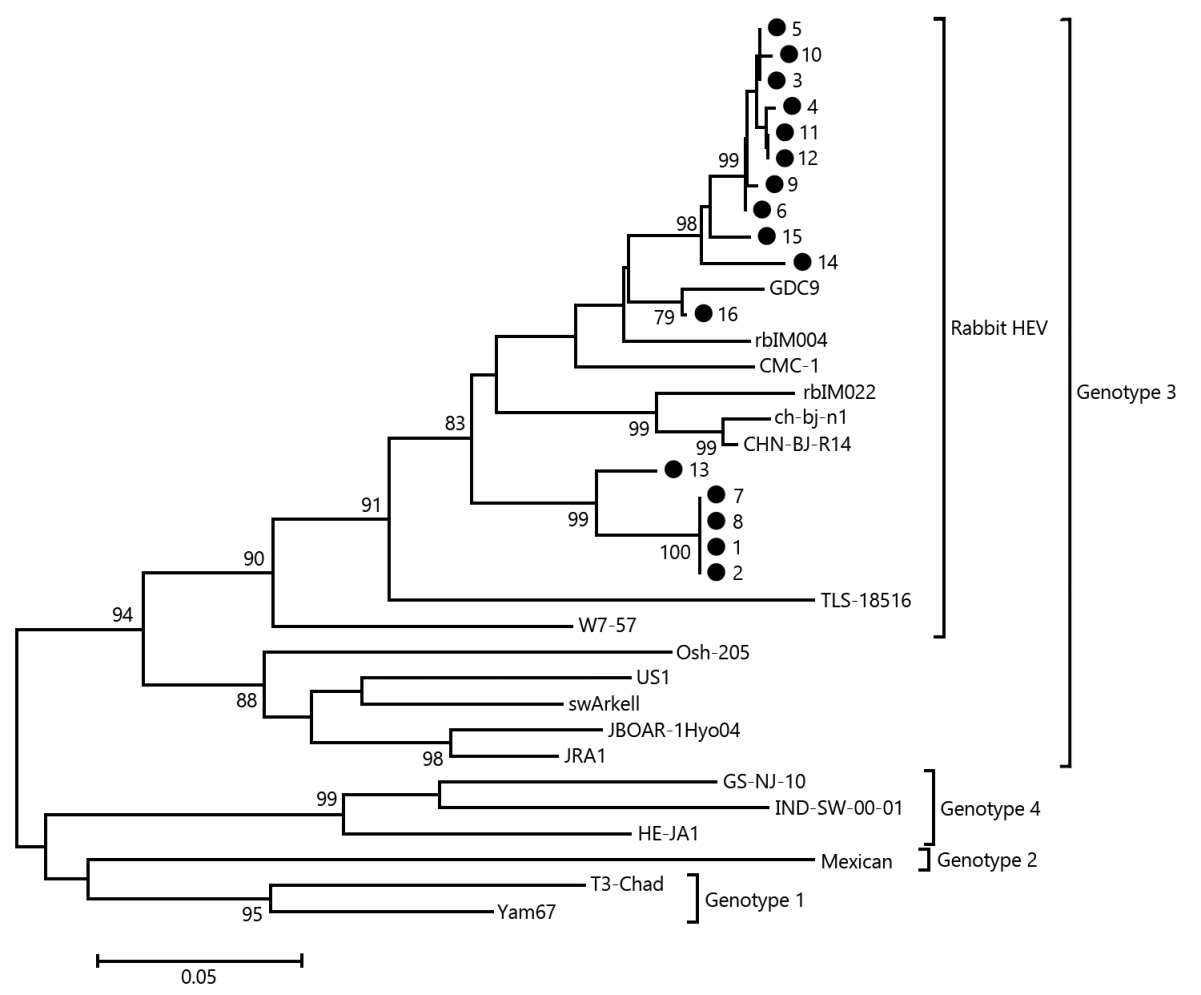
Phylogenetic analysis of hepatitis E virus (HEV) isolates from specific pathogen-free (SPF) rabbits, China, 2012−2015. The phylogenetic tree was constructed by using the neighbor-joining method, a partial nucleotide sequence of the open reading frame 2 region, and reported HEV sequences in GenBank as references. One thousand resamplings of the data were used to calculate percentages (values along branches) of tree branches obtained. Black circles indicate SPF rabbit isolates obtained during this study. GenBank accession numbers of all reference sequences (in parentheses) are FJ906895 (GDC9), AB740222 (rbIM004), JX565469 (CMC-1), AB740221 (rbIM022), GU937805 (ch-bj-n1), JX121233 (CHN-BJ-R14), JQ013793 (TLS-18516), JQ013792 (W7-57), AF455784 (Osh-205), AF060668 (US1), AY115488 (swArkell), AB189070 (JBOAR-1Hyo04), AP003430 (JRA1), JF309217 (GS-NJ-10), AY723745 (IND-SW-00-01), AB097812 (HE-JA1), M74506 (Mexican), AY204877 (T3-Chad), and AF459438 (Yam67). Scale bar indicates nucleotide substitutions per site.

Several cases of latent infection and seroconversion were observed (Table 1). Latency was defined as detection of HEV RNA or antibodies against HEV after negative results were observed during the first week in 4-week observation period. In batch no. 10, we found that 5, 3, and 1 SPF rabbits began excretion of HEV in stool during the second, third, and fourth weeks, respectively. Two rabbits seroconverted to antibodies against HEV during the second and third weeks.

## Conclusions

In our survey of 3 types of SPF laboratory animals commonly used for HEV studies in China, we detected previous HEV infection in all 3 types of animals, despite having purchased these animals from qualified vendors. We also detected HEV RNA in SPF rabbits, which suggested ongoing virus circulation in these animals. These findings emphasize the need for HEV screening of laboratory animals, not only for persons studying HEV but also for persons studying other pathogens, because the effects of co-infection are unknown. Before experiments are conducted, laboratory animals should be monitored for >2 weeks to ensure that no latent HEV infection is present.

Another concern is risk for zoonotic infection for in research personnel. HEV-3 and HEV-4 infect humans and other animals, and rabbit HEV-3 can infect cynomolgus macaques (*4*) and possibly humans (*5*). Therefore, safety of any research personnel who handle laboratory rabbits or pigs is a primary concern. Personal precautions should be fully implemented in the work environment.

HEV 239 vaccine is available in China, and studies have shown that this vaccine provided sustained protection against hepatitis E for <4.5 years (*9**,**10*). Thus, persons in China who have occupational exposure to HEV might benefit from vaccination.

This study has potential limitations. First, our results were determined only for 3 types of laboratory animals. Thus, sampling size should be enlarged to include other types of animals. Second, seroprevalence of antibodies against HEV was not determined for personnel who have close contact with these laboratory animals. Thus, risk for occupational transmission was not assessed. Future studies are warranted to address these issues.

In summary, our findings highlight the need for screening for HEV in laboratory animals. This screening will ensure experimental accuracy and prevent possibly zoonotic transmission of HEV to research personnel.

Technical Appendix Additional information on hepatitis E virus in 3 types of laboratory animals, China, 2012–2015.
