# Supplementary material for: Hepatitis E Virus in 3 Types of Laboratory Animals, China, 2012–2015
Source: Emerg Infect Dis. 2016 Dec;22(12):2157–9. doi: 10.3201/eid2212.160131 (PMC5189131; doi:10.3201/eid2212.160131)
Supplement: Technical Appendix — Additional information on hepatitis E virus in 3 types of laboratory animals, China, 2012–2015. [file 16-0131-Techapp-s1.pdf]

Article DOI: http://dx.doi.org/10.3201/eid2212.160131

Hepatitis E Virus in 3 Types of Laboratory Animals, China, 2012–2015

Technical Appendix

Technical Appendix Table 1. Microbes excluded in specific–pathogen-free animals

Animal	Microbes
Rabbits	*Salmonella* spp., *Listeria monocytogenes*, *Yersinia pseudotuberculosis*, *Y. enterocolitica*, pathogenic dermal fungi, *Pasturella multocida*, *Clostridium piliforme*, *Pasturella pneumotropica*, *Klebsiella pneumoniae*, *Staphylococcus aureus*, *Streptococcus pnemoniae*, β-hemolytic *Streptococcus*, *Pseudomonas aeruginosa*, rabbit hemorrhagic disease virus, Sendai virus, rotavirus, ectoparasites, *Toxoplasma gondii*, *Encephalitozoon cuniculi*, *Eimaria* spp., *Pneumocystis carinii*, all helminths and flagellates
Pigs	Foot and mouth disease virus, classical swine fever virus, porcine reproductive and respiratory syndrome virus, Japanese encephalitis virus, *Brucella* spp., pathogenic dermal fungi, *Leptospira* spp., pseudorabies virus, *Serpulina mahyodysenteriae*, *Bordetella bronchiseptica*, *Pasteurella multocida*, *Mycoplasma hyopneumoniae*, porcine parvovirus, porcine circovirus type 2, porcine transmissible gastroenteritis virus, swine vesicular disease virus, *Actinobacillus pleuropeumoniae*, *Salmonella* spp., *Streptococcus suis* type 2, ectoparasites, *Toxoplasma gondii*, coccidians, enterohelminths
Monkeys	*Salmonella* spp., pathogenic dermal fungi, *Shigella* spp., *Mycobacterium tuberculosis*, *Yesinia enterocolitica*, *Campylobacter jejuni*, Cercopithecidae herpesvirus type 1, simian retrovirus D, simian immunodeficiency virus, simian T lymphotropic virus type 1, simian pox virus, ectoparasites, *Toxoplasma gondii*, all helminths, *Entamoeba* spp., *Plasmodium* spp., flagellates

Technical Appendix Table 2. Studies reporting antibodies against hepatitis E virus and hepatitis E RNA in rabbits, by location and year*

Type, country	Year (reference)	Region	Sample	No. samples	Positive for antibodies against HEV, %	Positive for HEV RNA, %
Farmed or wild						
China	2009 (*1*)	Gansu	Serum	335	57.0	7.5
	2011 (*2*)	Beijing	Serum and feces	234	54.62	6.96
	2011 (*3*)	Multiple regions	Serum	1,094	15.4	1.6
	2012 (*4*)	Inner Mongolia	Serum	297	57.3†	71.6
	2013 (*5*)	Jiangsu	Bile and serum	928	NA	6.4
	2013 (*6*)	Hebei	Serum	289	23.2	10
	2014 (*7*)	Jiangsu	Feces	299	NA	5.0
	2015 (*8*)	Multiple regions	Feces	193	NA	1.2
United States	2011 (*9*)	California	Serum and feces	85	36‡	22
France	2012 (*10*)	Western region	Bile (farmed)	200	NA	7
	2010 (*10*)	Western region	Liver (wild)	205	NA	23
Italy‡	2015 (*11*)	Torino	Serum and liver	1	NA	NA
Germany§	2016 (*12*)	Greifswald	Serum	13	31	7.7
SPF						
United States	2014 (*13*)	Louisiana	Serum	10	50‡	NA
China¶	2015	Beijing	Serum and feces	332	7.5	4.8

*HEV, hepatitis E virus; NA, not applicable; SPF, specific pathogen free.
†Tested for IgG.
‡Only 1 rabbit was tested.
§Samples were collected in 1989.
¶Present study.

References

1. Zhao C, Ma Z, Harrison TJ, Feng R, Zhang C, Qiao Z, et al. A novel genotype of hepatitis E virus prevalent among farmed rabbits in China. J Med Virol. 2009;81:1371–9. PubMed http://dx.doi.org/10.1002/jmv.21536

2. Geng J, Wang L, Wang X, Fu H, Bu Q, Zhu Y, et al. Study on prevalence and genotype of hepatitis E virus isolated from Rex rabbits in Beijing, China. J Viral Hepat. 2011;18:661–7. PubMed http://dx.doi.org/10.1111/j.1365-2893.2010.01341.x

3. Geng Y, Zhao C, Song A, Wang J, Zhang X, Harrison TJ, et al. The serological prevalence and genetic diversity of hepatitis E virus in farmed rabbits in China. Infect Genet Evol. 2011;11:476–82. PubMed http://dx.doi.org/10.1016/j.meegid.2010.12.012

4. Jirintai S, Jinshan, Tanggis, Manglai D, Mulyanto, Takahashi M, et al. Molecular analysis of hepatitis E virus from farm rabbits in Inner Mongolia, China and its successful propagation in A549 and PLC/PRF/5 cells. Virus Res. 2012;170:126–37. PubMed http://dx.doi.org/10.1016/j.virusres.2012.09.015

5. Wang S, Dong C, Dai X, Cheng X, Liang J, Dong M, et al. Hepatitis E virus isolated from rabbits is genetically heterogeneous but with very similar antigenicity to human HEV. J Med Virol. 2013;85:627–35. PubMed http://dx.doi.org/10.1002/jmv.23504

6. Geng Y, Zhang H, Li J, Huang W, Harrison TJ, Zhao C, et al. Comparison of hepatitis E virus genotypes from rabbits and pigs in the same geographic area: No evidence of natural cross-species transmission between the two animals. Infect Genet Evol. 2013;13:304–9. PubMed http://dx.doi.org/10.1016/j.meegid.2012.11.006

7. Han J, Zeng H, Wang L, Liu P, Liu L, Xia J, et al. Hepatitis E virus infection in farmed rabbits and swine in the eastern Chinese city Lianyungangshowing no potential interspecies transmission. J Med Virol. 2014;86:1898–904. PubMed http://dx.doi.org/10.1002/jmv.24003

8. Xia J, Zeng H, Liu L, Zhang Y, Liu P, Geng J, et al. Swine and rabbits are the main reservoirs of hepatitis E virus in China: detection of HEV RNA in feces of farmed and wild animals. Arch Virol. 2015;160:2791–8. PubMed http://dx.doi.org/10.1007/s00705-015-2574-0

9. Cossaboom CM, Cordoba L, Dryman BA, Meng XJ. Hepatitis E virus in rabbits, Virginia, USA. Emerg Infect Dis. 2011;17:2047–9. PubMed http://dx.doi.org/10.3201/eid1711.110428

10. Izopet J, Dubois M, Bertagnoli S, Lhomme S, Marchandeau S, Boucher S, et al. Hepatitis E virus strains in rabbits and evidence of a closely related strain in humans, France. Emerg Infect Dis. 2012; 18:1274–81. PubMed

11. Caruso C, Modesto P, Prato R, Scaglione FE, De Marco L, Bollo E, et al. Hepatitis E virus: first description in a pet house rabbit.a new transmission route for humans? Transbound Emerg Dis. 2015;62:229–32. PubMed http://dx.doi.org/10.1111/tbed.12348

12. Eiden M, Vina-Rodriguez A, Schlosser J, Schirrmeier H, Groschup MH. Detection of hepatitis E virus in archived rabbit serum samples, Germany 1989. Food Environ Virol. 2016;8:105–7. PubMed http://dx.doi.org/10.1007/s12560-015-9222-4

13. Birke L, Cormier SA, You D, Stout RW, Clement C, Johnson M, et al. Hepatitis E antibodies in laboratory rabbits from 2 US vendors. Emerg Infect Dis. 2014;20:693–6. PubMed http://dx.doi.org/10.3201/eid2004.131229
